# Systematic comparative validation of self-report measures of sedentary time against an objective measure of postural sitting (activPAL)

**DOI:** 10.1186/s12966-018-0652-x

**Published:** 2018-02-26

**Authors:** S. F. M. Chastin, M. L. Dontje, D. A. Skelton, I. Čukić, R. J. Shaw, J. M. R. Gill, C. A. Greig, C. R. Gale, I. J. Deary, G. Der, P. M. Dall, on behalf of the Seniors USP team, Dawn A. Skelton, Sebastien Chastin, Simon Cox, Elaine Coulter, Iva Čukić, Philippa Dall, Ian Deary, Geoff Der, Manon Dontje, Claire Fitzsimons, Catharine Gale, Jason Gill, Malcolm Granat, Cindy Gray, Carolyn Greig, Elaine Hindle, Karen Laird, Gillian Mead, Nanette Mutrie, Victoria Palmer, Ratko Radakovic, Naveed Sattar, Richard Shaw, John Starr, Sally Stewart, Sally Wyke

**Affiliations:** 1 Institute for Applied Health Research, School of Health and life Science, Glasgow Caledonian University, Cowcaddens Road, Glasgow, G4 0BA Scotland, UK; 2 Department of Movement and Sports Sciences, Faculty of Medicine and Health Science, Ghent University, Ghent, Belgium; 3 School of Population and Global Health, University of Western Australia, Perth, Australia; 4 Centre for Cognitive Ageing & Cognitive Epidemiology, Department of Psychology, University of Edinburgh, Edinburgh, UK; 5 MRC/CSO Social and Public Health Sciences Unit, University of Glasgow, Glasgow, UK; 6 Institute of Cardiovascular and Medical Sciences, University of Glasgow, Glasgow, UK; 7 School of Sport, Exercise and Rehabilitation Sciences and MRC-Arthritis Research UK Centre for Musculoskeletal Ageing and Health, University of Birmingham, Birmingham, UK; 8 MRC Lifecourse Epidemiology Unit, University of Southampton, Southampton, UK

**Keywords:** Sitting, Physical activity, Surveillance, Sedentary behaviour, Validation, Questionnaires, activPAL, Measurement

## Abstract

**Background:**

Sedentary behaviour is a public health concern that requires surveillance and epidemiological research. For such large scale studies, self-report tools are a pragmatic measurement solution. A large number of self-report tools are currently in use, but few have been validated against an objective measure of sedentary time and there is no comparative information between tools to guide choice or to enable comparison between studies. The aim of this study was to provide a systematic comparison, generalisable to all tools, of the validity of self-report measures of sedentary time against a gold standard sedentary time objective monitor.

**Methods:**

Cross sectional data from three cohorts (*N* = 700) were used in this validation study. Eighteen self-report measures of sedentary time, based on the TAxonomy of Self-report SB Tools (TASST) framework, were compared against an objective measure of postural sitting (activPAL) to provide information, generalizable to all existing tools, on agreement and precision using Bland-Altman statistics, on criterion validity using Pearson correlation, and on data loss.

**Results:**

All self-report measures showed poor accuracy compared with the objective measure of sedentary time, with very wide limits of agreement and poor precision (random error > 2.5 h). Most tools under-reported total sedentary time and demonstrated low correlations with objective data. The type of assessment used by the tool, whether direct, proxy, or a composite measure, influenced the measurement characteristics. Proxy measures (TV time) and single item direct measures using a visual analogue scale to assess the proportion of the day spent sitting, showed the best combination of precision and data loss. The recall period (e.g. previous week) had little influence on measurement characteristics.

**Conclusion:**

Self-report measures of sedentary time result in large bias, poor precision and low correlation with an objective measure of sedentary time. Choice of tool depends on the research context, design and question. Choice can be guided by this systematic comparative validation and, in the case of population surveillance, it recommends to use a visual analog scale and a 7 day recall period. Comparison between studies and improving population estimates of average sedentary time, is possible with the comparative correction factors provided.

**Electronic supplementary material:**

The online version of this article (10.1186/s12966-018-0652-x) contains supplementary material, which is available to authorized users.

## Background

Societal changes have made sitting the dominant posture in many situations of daily living such as at school, at work, while travelling and during many leisure time activities. The amount of time we spend sitting everyday has increased over the last 50 years, and is forecast to continue increasing [1]. Over the last two decades, this has become a matter of concern in public health. Under the umbrella term of sedentary behaviours (SB) [2, 3], time spent sitting is associated with poorer health outcomes, chronic diseases and premature mortality [4]. Several nations have issued specific recommendations to reduce sedentary time as part of their physical activity guidelines and policy [5, 6]. Adequate surveillance systems and large scale epidemiological studies are required to monitor sedentary time and evaluate its impact on populations. These need accurate and valid measures of sedentary time. Objective sensors of posture provide the most valid and accurate measures of sedentary time, but self-report measures are more pragmatic, as they are generally cheaper and more easily integrated in existing surveillance or epidemiological studies [7]. A recent systematic inventory of existing self-report measures of sedentary time reviewed their measurement characteristics [8] and revealed that:

1. there was a large number (*n* = 37) of different measures of sedentary time currently available for use in adult populations;
2. very few of these (*n* = 4) had been validated against an appropriate objective reference measure;
3. there was no comparative information to guide choice of the best measure to use and how to compare results from different studies using different tools;
4. there was a general consensus that the accuracy and validity of these tools needed to be improved, but it was not known which characteristics of the tools required modification to improve accuracy and validity.

The aim of the current study was to evaluate the accuracy, precision, criterion validity and data loss of self-report measures of sedentary time against an adequate objective measure of sedentary time, using a systematic process to provide comparison that is generalizable to self-report tools and to allow harmonisation of existing data.

## Methods

### Study design and sample

**Fig. 1:**
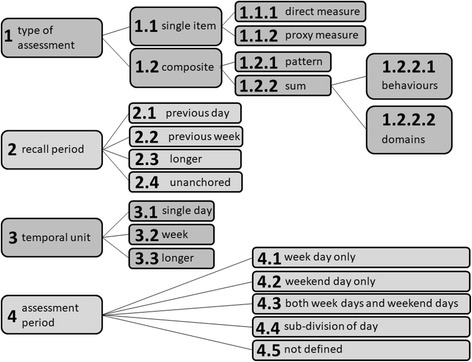
The TASTT taxonomy (reproduced from [8])

The study is a validation study of self-report measures of sedentary time based on the TAxonomy of Self-report Sedentary behaviour Tools (TASST) framework [8] (Fig. 1) to provide a systematic comparison process. Currently there are at least 37 different self-report tools for measuring sedentary time in adults, representing at least 140 individual questions [8]. Attempting to provide comparative validation for all of them would be pragmatically very difficult because of burden on respondent and cost, and limit the results to information about existing tools only. Instead, a systematic approach was used which allowed testing and validation of specific characteristics of self-report tools and the assessment of how the combinations of these characteristics influenced measurement characteristics. It was considered more useful to derive information about the taxonomic characteristics that could then be generalised. Eighteen self-report measures of sedentary time, representing combinations of the two main domains of the TASST framework, were validated against an objective measure of sedentary time. Eighteen self-report measures of sedentary time representing all relevant branches of TASST were validated against an objective measure of sedentary time. Cross-sectional data were gathered in three Scottish cohorts of older adults; the Lothian Birth Cohort 1936 (LBC1936) [9], and the West of Scotland Twenty-07 study (T07) 1930s and 1950s birth cohorts [10]. The current study is part of the MRC funded Seniors USP (Understanding Sedentary Pattern) project, which aims to understand determinants of sedentary behaviour. For the Seniors USP project a total of 1757 cohort members (*n* = 524 LBC1936; *n* = 1233 T07) were invited to participate in the study. This study was embedded within Wave 4 of the LBC1936 cohort study but ran as a separate data collection wave for T07.

### Subjective measurement of sedentary behaviour

Eighteen self-report measures of sedentary time were constructed, based on the TASTT framework, to provide a representative sample of characteristics in current and future self-report tools of sedentary time for use in an adult population. The selection focused on the first two domains of TASTT, namely the type of assessment and the recall period. These two domains cover the majority of the variations found in self-report measures and are most likely to influence the measurement characteristics [8] .

**Fig. 2:**
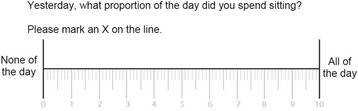
Example of self-report assessment of the proportion of the day spent sitting using a visual analogue scale (VAS)

The systematic selection covered five different types of assessment (Taxon 1):

- two single item direct measures of sedentary time (Taxon 1.1.1), where the term direct refers to the TASST nomenclature for measures that ask individuals to recall total sedentary time directly (as opposed to using a proxy measure). This should not be confused with the term direct often used to refer to objective measure of sedentary time.
- a direct question about the total time spent sitting;
- a visual analogue scale of the proportion of the day spent sitting (Fig. 2);
- a single item proxy measure (TV time) (Taxon 1.1.2)
- a composite measure based on pattern (Taxon 1.2.1) asking respondents to report the number of bouts of sitting and their average duration (multiplied together to get total duration);
- a composite measure based on the sum of behaviours (Taxon 1.2.2.1) asking respondents to report the time spent in 13 specific SBs (extracted from [3]): watching TV, work, using computer/screen for leisure, reading, listening or playing music, engaging in seated hobbies, talking, eating, self-care, performing activities of daily living, napping, sitting in transport and sitting during leisure activities outside the home (e.g. watching a play at the theatre)); and
- a composite measure based on the sum of domains (Taxon 1.2.2.2) asking respondents to report the time spent in four different domains (extracted from [3]): work, home, transport and leisure).

Each of these types of measure was applied across three different recall periods (Taxon 2); previous day recall, previous week recall (sometimes called 7-day recall), and unanchored recall (respondents are asked about their usual behaviour without reference to the present, for example a usual week).

All self-report measures also used a temporal unit (Taxon 3) of a day, asking for time spent sitting on a day within the recall period. The assessment period (Taxon 4) was not defined, meaning that all days within the recall period were considered. The actual questions used are available in the Additional file 1.

### Objective reference measure

Self-report tools were validated against the same reference measure; average daily sedentary time derived from seven full days of objective measurement. Sedentary time was measured objectively using an activPAL activity monitor (activPAL3c, PAL Technologies Ltd., UK). This small (53 × 35 × 7 mm) and light (15 g) tri-axial activity monitor is worn on the anterior thigh. The monitor samples acceleration at 20 Hz, which is then categorised into time spent in sedentary or upright posture based on thigh inclination. Monitors were waterproofed so participants could wear the monitor for the full measurement period without having to remove and reattach it. They were heat-sealed inside plastic tubing (layflat tubing, Packaging Aids Ltd., UK), and attached by a researcher to the dominant leg using a hypoallergenic double sided adhesive pad (PAL stickies, PAL Technologies Ltd., UK) and covered by a waterproof dressing (Opsite Flexifix, Smith & Nephew, UK). Besides measuring the number of steps and number of sit-to-stand transitions per day, the activPAL classifies activity behaviour into time spent sitting/lying, standing, and walking. The activPAL is valid for step count, time spent sedentary (sitting and lying), standing and walking, and is currently regarded as a gold standard for the objective measurement of sedentary/sitting time and pattern of SB [11–14].

As the activPAL monitor is unable to distinguish between sitting/lying and sleeping, participants were asked to keep a diary reporting the time they fell asleep the previous night and the time they woke up for each day of monitoring.

### Procedure

Participants received the first questionnaire at home via post, containing questions about sedentary time for the unanchored recall period. Participants were then met on two occasions. The first visit took place at a clinical research facility for the LBC1936 cohort and at home for the T07 cohort. During this visit the unanchored SB questionnaire was retrieved and a researcher attached the activPAL activity monitor. Participants were asked to wear the activPAL for at least 7 full days continuously including overnight and during bathing/swimming, while continuing their normal daily activities. Day one was the first day of continuous activity monitoring and corresponded to the day after the first visit. During the first visit, the participants were given the two remaining questionnaires. The first questionnaire, asking about sedentary time on the previous day, was completed on day 3 (about SB on day 2) of the monitoring period. The second questionnaire, asking about sedentary time during the previous week, was completed on day 8 (covering the period of day 1 to 7). After day 8, the participants came back to the research facility, or the researcher visited the participants at home, where the researcher removed the activPAL activity monitor and collected and checked the questionnaires.

### Cohort data

Demographic information about the participants was obtained either through primary data collection as part of the Seniors USP project, or from the most recent wave of cohort data at which it was collected. Age was reported as an average for each cohort (one LBC1936 cohort and two age cohorts from the T07 study). Gender, socio-economic status (lifetime social class based on highest achieved household occupation, stratified as high (professional, managerial) or low (skilled non-manual, skilled manual, semi-skilled and unskilled), and highest level of education were obtained from previously-collected cohort data. Self-reported health limitations were measured concurrently (categorised as no long standing illness, a long standing illness that doesn’t affect activities, and a long-standing illness that does affect activities). Body mass index (BMI) was calculated from weight measured concurrently, and height measured at last cohort wave.

### Data processing

Data were downloaded from the monitor using the activPAL proprietary software (version 7.2.32, PAL Technologies Ltd., UK). Participants with less than seven full 24 h days of activPAL data, or with incomplete sleep diaries, were excluded, in order to avoid making any assumptions about wear time. The statistical programming environment and language R (6) was used to combine the activPAL and sleep diary data to compute waking day sedentary time and number of sedentary bouts for each 24 h period (from midnight) and to perform all the analyses. Objective total sedentary time and number of sedentary bouts were calculated as the mean daily recorded sedentary time and the mean daily number of sedentary bouts over the seven days. Similarly, the proportion of the day spent sedentary was computed as the average over seven days of the daily proportion of sedentary time during waking hours.

Outcomes from all self-report measures had a temporal unit of a day, thus the value reported by the participant was used directly as the value of sedentary time per day in each category. Total sedentary time for the composite self-report measures was calculated as a sum of domains (4 items), or a sum of behaviours (13 items). Finally, for the composite pattern measure, the number of bouts was multiplied by the average sedentary time per bout.

### Analysis

The amount of data lost because participants did not answer the self-reported sedentary time questions or provided unreadable or obviously incorrect answers (e.g. more than 24 h of total sedentary time per day) was used to provide an indication of ease of use. Self-report SB was compared with objective SB using Bland-Altman plots and statistics to assess agreement. ActivPAL data were treated as “gold standard” and the Bland and Altman plots computed using Krouwer’s method [15]. Pearson correlation was used to assess criterion validity. The mean difference between objective data and self-report measures was used to gauge the accuracy of the self-report measure (systematic error). This value can also be used as a correction factor to be added to values obtained with a specific self-report tool in the right taxon in order to gain more accurate estimates sedentary time. The standard deviation of the difference between objective data and self-report measures was used to gauge the precision of the self-report measure (random error).

## Results

### Response and sample descriptive statistics

Data were pooled from all three cohorts. Seven hundred and seventy three participants agreed to wear the activPAL; of those, 700 provided seven full days of data and were included in this analysis. Cohort ages were 64 (T07 1950s cohort, *n* = 310), 79 (LBC1936, *n* = 271), and 83 (T07 1930s cohort, *n* = 119). There were slightly more women (*n* = 361, 52%) than men. Participants had a mean BMI of 27.6 ± 4.5 kgm^− 2^ (range 17.0 to 50.5), with the majority from higher socioeconomic classes (*n* = 422, 60%), and 206 (29%) achieved degree level of education. About half the participants (*n* = 327, 47%) reported they had no long-standing health conditions, and only 160 (23%) reported their long-standing health condition limited their life.

Using the objective measures, participants spent on average 10.5 (± SD 2.0) hours [62.6 ± 10.9% of waking day] sitting, and engaged on average in 46.1 (± SD 13.2) sedentary bouts per day. Sample average sedentary time and number of sedentary bouts for each of the self-report methods are reported in Table 1.

**Table 1:** Sample sedentary time measured objectively (activPAL) and self-report measures of sedentary time. Single (total sitting/proportion), Proxy (TV, screen), Composite (behaviour, domain, pattern)

Measure of SB		Taxon	Mean	Standard deviation	Median	25–75 percentile
Objective measure						
	Total sitting time (min/day)		628.7	120.7	625.5	545.9–707.7
	Proportion of waking day spent sitting (%)		62.6	10.9		
Self reported						
Previous day recall						
	*Single item* total sedentary time (min/day)	1.1.1/2.1	422.5	160.9	420.0	300.0–525.0
	*Single item* Proportion of the day (%) [min/day]	1.1.1/2.1	49.8 [478.1]	17.5 [168.0]	50.0 [480.0]	37.1–60.0 [357.0–576.0]
	*Single item proxy* TV time (min/day)	1.1.2/2.1	191.5	123.2	180.0	120.0–270.0
	*Composite* Sum of domains (min/day)	1.2.2.2/2.1	499.0	196.8	480.0	360.0–600.0
	*Composite* Sum of behaviours (min/day)	1.2.2.1/2.1	811.9	331.5	770.0	600.0–975.0
	*Composite* Pattern (min/day)	1.2.1/2.1	589.8	587.3	450.0	270.0–718.0
Previous week recall						
	*Single item* total sedentary time (min/day)	1.1.1/2.2	486.0	486.5	420.0	300.0–491.3
	*Single item* Proportion of the day (%) [min/day]	1.1.1/2.2	50.9 [488.6]	16.2 [155.5]	50 [480.0]	40.0–60.0 [384–576]
	*Single item proxy* TV time (min/day)	1.1.2/2.2	235.9	206.5	200.0	126.3–288.8
	*Composite* Sum of domains (min/day)	1.2.2.2/2.2	662.6	466.5	570.0	440.0–750.0
	*Composite* Sum of behaviours (min/day)	1.2.2.1/2.2	1125.2	867.8	930.0	713.8–1260.0
	*Composite* Pattern (min/day)	1.2.1/2.2	845.4	1796.7	480.0	300.0–900.0
Unanchored (usual day)						
	*Single item* total sedentary time (min/day)	1.1.1/2.4	379.2	152.3	360.0	270–960
	*Single item* Proportion of the day (%) [min/day]	1.1.1/2.4	46.6 [447.4]	18.1 [173.8]	45.0	30.0–60.0 [432.0–576.0]
	*Single item proxy* TV time (min/day)	1.1.2/2.4	215.8	123.6	180.0	120.0–270.0
	*Composite* Sum of domains (min/day)	1.2.2.2/2.4	557.4	234.0	540.0	415.0–662.5
	*Composite* Sum of behaviours (min/day)	1.2.2.1/2.4	933.8	428.6	882.5	670.0–1110.0
	*Composite* Pattern (min/day)	1.2.1/2.4	266.7	743.9	64.0	25.0–150.3

Single item proportion of the day was converted into sitting time per day in minutes assuming a standard 16 h waking day

### Validation

**Fig. 3:**
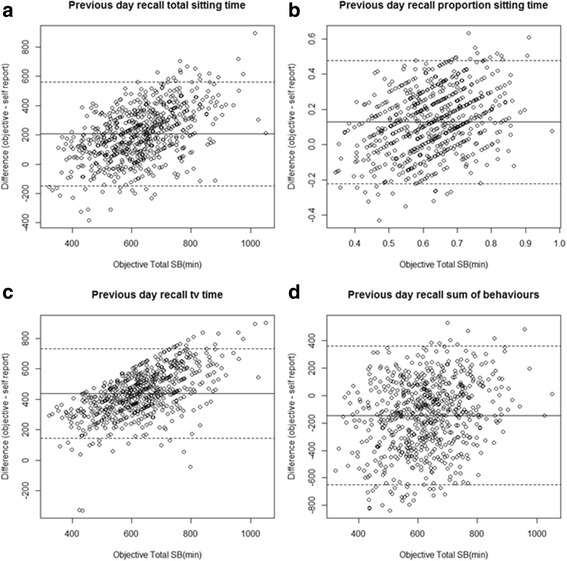
Bland and Altman plot comparing: **a** direct measure of total sitting time, **b** direct measure using a visual analogue scale, **c** direct measure using a proxy measure (TV time) and **d** composite measure based on sum of behaviours for a previous day recall with the average recorded daily sitting time recorded with activPAL

For each of the 18 self-report measures, results from Bland-Altman statistics including the bias in mean and limits of agreement (LOA), standard deviation of the difference, percentage of data loss, and Pearson correlation coefficient and *p*-values are reported in Table 2. Bland and Altman plots are presented in Fig. 3 for the direct measure of total sedentary time, direct measure using a visual analogue scale, proxy measure (TV time) and composite measure based on sum of behaviours for a previous day recall. All other plots are in the Additional file 2.

**Table 2:** Bland and Altman (B-A) statistics (differences objective – self-report measure) including bias, lower and upper limits of agreement (LOA), percentage of data loss, and correlation statistics results of the comparison between self-report measures of sedentary time s against objective data (activPAL) organised according to the TASST taxonomy

Self reported measures	TASST Taxon (Fig. 1)	% data loss	B-A bias Correction factor (min) [%]	B-A Lower LOA (min) [%]	B-A Upper LOA (min) [%]	St. dev of difference (min) [%]	correlation	*p*-value
Previous day recall								
*Single item* total sedentary time (min)	1.1.1/2.1	1.0	207.0	− 146.8	560.8	180.5	0.20	< 0.001
*Single item* Proportion of the day (%) [min]	1.1.1/2.1	0.7	12.9 [123.8]	−22.1 [−212.2]	47.8 [458.9]	17.8 [170.9]	0.28	< 0.001
*Single item proxy* TV time (min)	1.1.2/2.1	2.4	439.0	144.8	733.2	150.1	0.24	< 0.001
*Composite* Sum of domains (min)	1.2.2.2/2.1	0.7	130.3	− 272.5	533.1	205.5	0.23	< 0.001
*Composite* Sum of behaviours (min)	1.2.2.1/2.1	4.3	− 145.2	−651.2	366.7	258.2	0.23	< 0.001
*Composite* Pattern (min)	1.2.1/2.1	9.7	138.0	− 471.6	747.5	311.0	0.17	< 0.001
Previous week recall								
*Single item* total sedentary time (min)	1.1.1/2.2	4.9	221.3	− 149.9	563.9	182.1	0.23	< 0.001
*Single item* Proportion of the day (%) [min]	1.1.1/2.2	0.9	11.8 [113.2]	−19.3 [− 185.3]	42.9 [411.8]	15.9 [152.6]	0.36	< 0.001
*Single item proxy* TV time (min)	1.1.2/2.2	2.6	406.2	80.6	731.9	166.1	0.23	< 0.001
*Composite* Sum of domains (min)	1.2.2.2/2.2	3.9	34.5	− 412.9	482.1	228.3	0.30	< 0.001
*Composite* Sum of behaviours (min)	1.2.2.1/2.2	16.9	− 244.9	− 755.2	265.3	260.3	0.32	< 0.001
*Composite* Pattern (min)	1.2.1/2.2	14.6	99.1	− 528.8	726.9	320.3	0.23	< 0.001
Unanchored (usual day)								
*Single item* total sedentary time (min)	1.1.1/2.4	2.4	250.6	− 135.1	549.2	174.6	0.20	< 0.001
*Single item* Proportion of the day (%) [min]	1.1.1/2.4	2.3	16.1 [154.6]	−18.9 [− 181.4]	51.1 [490.6]	17.9 [171.8]	0.32	< 0.001
*Single item proxy* TV time (min)	1.1.2/2.4	1.9	415.0	124.7	705.4	148.1	0.26	< 0.001
*Composite* Sum of domains (min)	1.2.2.2/2.4	1.9	77.9	− 372.8	528.8	230.0	0.16	< 0.001
*Composite* Sum of behaviours (min)	1.2.2.1/2.4	9.1	− 219.8	− 725.3	285.6	257.9	0.33	< 0.001
*Composite* Pattern (min)	1.2.1/2.4	7.1	472.9	−33.8	979.6	258.5	0.02	0.624

Single item proportion of the day was converted into sitting time per day in minutes assuming a standard 16 h waking day

### Accuracy

The Bland and Altman statistics show that the differences in sample mean measured by the self-report variable from the objective measure were often large, ranging from a 473 min underestimation for a composite measure based on pattern and an unanchored recall period, to a 245 min overestimation for a composite measure based on sum of behaviours and a previous week recall period. With the exception of composite measures based on sum of behaviours, all of the self-report measures underestimated time spent sitting. In general, the type of assessment was more of an influence on size of bias than the recall period. Within each recall period, composite measures based on sum of domains consistently had the lowest bias.

### Precision

The Bland and Altman plots (Fig. 3 and Additional file 2) show that the limits of agreement were very wide (Table 2) ranging from around 600 min to 1000 min. Proxy measures, apart from a few cases, consistently underestimated total sedentary time. For all other self-report measures the error could be in either direction (under or over reported). However, there were marked trends, with error decreasing proportionally to sedentary time in several self-report measures (Fig. 3 and Additional file 2). For evaluation of total sedentary time using a single question there was a tendency for individuals engaging in less sedentary time to over report and those engaging in more sedentary time to under report.

The biggest influence on the precision of self-report measures (standard deviation of the difference) was the type of assessment used, with recall period having less influence. Across all recall periods, the proxy measure (TV time) and the assessment with a visual analogue scale of the proportion of the day spent sedentary (single item direct measure of sitting) had the lowest standard deviation of the difference (around 160 min). Composite measures based on pattern and the sum of behaviours had by far the lowest precision of those assessed around 320 min and 260 min respectively.

### Correlation

Correlations of the self-report measure with the objective measure were all low, with correlation coefficients ranging from 0.02 to 0.36. Many of the self-report measures clustered around similar values; out of 21 measures, 13 (62%) were in the range 0.2 to 0.3, while 8 (28%) were either 0.23 or 0.24. Within each recall period, a single item based on proportion of the day and a composite measure based on sum of behaviours tended to have higher correlation with objective measures. Recall period did not appear to have a large influence on correlation; however, for those types of assessment with higher correlation coefficients, previous day recall tended to be worse than either previous week or unanchored, but that difference was not statistically significant. The composite measures based on pattern had a particularly poor correlation with objective measures. Self-reported number of sedentary bouts showed a significant correlation (*p* = 0.017) with the objective measure for only a previous day recall period, but this was very low (correlation coefficient of 0.05). This suggests that self-report number of sedentary bouts is unlikely to be valid measure.

### Data loss

The amount of data lost because participants did not answer the self-reported sedentary time questions or provided unreadable or obviously incorrect answers (e.g. sedentary time larger than 24 h) is reported as percentage in Table 2. Overall, the previous day recall period had a slightly lower percentage of data loss and the previous week recall period the highest. The lowest percentages of data loss were for a composite measure based on sum of domains with a previous day recall period and the single item assessment of the proportion of time sitting for both previous day and previous week recall periods. However, differences in data loss between most self-report measures were relatively small. The exceptions were the number of sedentary bouts, composite measures based pattern, and the sum of behaviours, which had consistently large proportion of data loss. The source of data loss was different between measures; most data loss for composite measures based on the sum of behaviours was due to obviously incorrect answers, whereas the pattern metric was more evenly balanced between data not reported and data that were obviously incorrect.

### Comparison between self-report measures

**Fig. 4:**
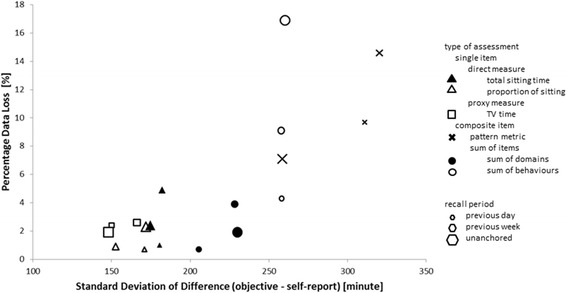
Comparative plot of data loss and random error in sedentary time for eighteen self-report measures

Overall accuracy is low and criterion validity is relatively similar for most tools. However precision and data loss show more salient differences. Figure 4 shows a comparative plot of the precision and percentage of data loss for the 18 self-report measures of sedentary time. This plot can be used to guide the selection of self-report measures and to identify characteristics of these measures influencing measurement. Eight of the 18 measures cluster with less than 4% of data loss and 180 min (3 h) of error. Amongst these, the most advantageous appear to be proxy measures (TV time) and direct measure of the proportion of daily sedentary time using a visual analogue scale, regardless of the recall period. Composite measures appear to be subject to more error. The amount of data loss and error grow with the complexity of the composite measure, either because of the number of items included (sum of behaviours) or difficulty in estimating the components (pattern).

## Discussion

This study presents a comparative validation of self-report measures of sedentary time and pattern against an objective measure of sitting, using a systematic process. The use of the TASST framework to construct a set of 18 self-report tools, enables generalization of the results to most existing self-report tools, and allows recommendations on future development of self-report tools to be made. Overall, self-report tools of total sedentary time show poor accuracy, with large bias and wide limits of agreement. With the exception of composite measures based on the sum of time spent in different SBs, all self-report measures under-reported sedentary time. This will affect surveillance systems and studies obtaining population estimates of average sedentary time. It also makes comparisons between surveys and studies using different self-report tools difficult. Using the correction factors shown in Table 2 to remove the systematic part of the error will provide more accurate estimates of population average sedentary time and enable better comparison between studies using different tools.

All self-report tools showed low correlation (0.38 in the best case) with objective data and low precision, with random error generally larger than 2.5 h. This will affect epidemiological studies that try to ascertain relationships between sedentary time and health outcomes or potential determinants. Surprisingly, proxy measures such as TV time performed the best in this respect. This might be because TV time is a ubiquitous SB that often follows a specific schedule, making it easier to recall. The only other measure of total sedentary time that provided comparable measurement characteristics with TV time, was an assessment of total sedentary time using a visual analogue scale of the proportion of the day spent sitting. Generally, composite measures were subject to more random error, which grows with the complexity of the measure. For example, recollecting time spent in thirteen different SBs leads to larger random error than recollecting time sent sedentary in four domains. Similarly, composite measures based on patterns of SB requires recollecting the number of bouts and the average duration of a sedentary bout. This appears both very imprecise, and difficult for participants to complete (high rate of data loss through missing data).

Finally, data loss due to the self-report measures either not being completed or not providing exploitable data is another important factor to consider. In this respect, most self-report tools had less than 5% data loss (Fig. 4). The tools most affected by data loss were composite measures, especially if they require recollecting more subscales or complex constructs such as pattern, that a participant might find difficult to consider.

The biggest influence of measurement characteristics appears to be the type of question asked, and not the recall period used. Recent reports [16–18] saw reducing the recall period as a positive way to improve the validity and accuracy of self-report measures for SB. In this study, recall period appears to have little (and not a systematic) influence on the accuracy, precision, criterion validity or data loss of self-report measures of SB. To improve measurement characteristics, the type of assessment seems a more promising feature to change.

The results show that assessment of pattern is least valid type of self-report of SB. Self-report assessment of the number of SB bouts is prone to very large systematic and random error and does not correlate with objective assessment. From studies using objective monitors, it is possible that the pattern in which ST is accumulated may influence health as well as the total time spent sedentary [19, 20]. However, it appears that self-report is not a valid measure of pattern of SB, which might preclude large scale studies of the impact of pattern of SB and effect of “breaks” using self-report measures.

### Recommendations

**Fig. 5:**
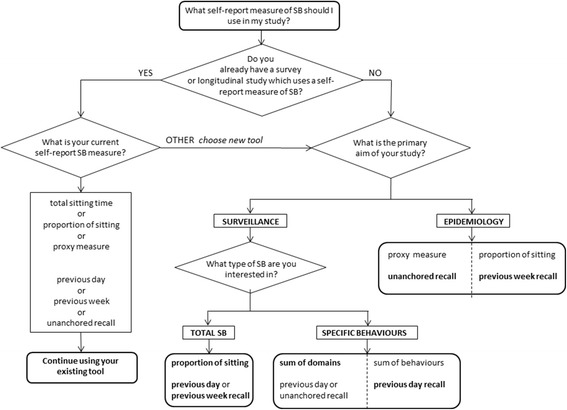
Decision flow chart for choice of self-report instrument to measure sedentary time

This comparative validation study clearly shows that no self-reported tool of sedentary time provides a measurement of sedentary time with the same accuracy, precision and validity of objective SB measures. Therefore, when possible, objective measures should be used instead of self-report tools. Using the results of this study, in conjunction with the TASTT framework, some recommendations can be made about choosing the best possible self-report tool to measure SB if an objective measure is not possible. These are summarised as a flow chart in Fig. 5 and will depend primarily on whether a survey or study already uses a pre-existing measure, and on the main aim of the study [21]. Recommendations are expressed here in terms of taxa within the TASST framework, but this can be translated to specific self report tools using Additional file 3 which provides a table mapping the existing tools identified in a previous review [8] against the TASST domains assessed in the current validation study. For an existing survey that already includes assessment of sedentary time, it is probably only worth changing this assessment if the tool used does not fall within the type of assessment covered by a single item (direct taxon 1.1.1 or proxy taxon 1.1.2) with any recall period, as any gain in precision or reduced data loss are likely to be small (Fig. 4). In this case, the continuity of data collected with previous samples/studies is probably more important than moderate improvements in future data collected, especially in terms of population surveillance. In the case of a new survey or study, or the first introduction of a sedentary time assessment, the choice should be guided by the aim of the survey or tool.

### Surveillance

If the primary aim is surveillance of total sedentary time, then using a visual analogue scale and either a previous day or previous week recall period would give the most accurate and precise results. However, if the aim of the surveillance is to look into more details at a specific SB or a specific domain where SB occurs, but an estimate of total sedentary time is still required, then composite measures should be adopted. A composite measure based on sum of domains would be preferable to one based on sum of behaviours for surveillance of total sedentary time, and should be used unless the aim of the survey or study is to monitor distribution of time between SBs. For this type of assessment, however, gains can be made in terms of reduced data loss by adopting short (previous day) or unanchored recall periods.

### Epidemiology

If the primary aim is epidemiological research, then strong correlation and low random error are the most important measurement characteristics to consider. Using a proxy measure with an unanchored recall period or adopting a visual analogue scale assessment of the proportion of the day spent sitting provide the most valid measures with the lowest data loss. These should be preferentially used over other types of assessment and recall periods. However, a recent consensus recent consensus highlight that understanding the context of sedentary time is a research priority [22]. In this case composite measure would be more appropriate. Choosing the appropriate recall period might avoid unwanted data loss (Fig. 4).

### Strengths and limitations

The strengths of this study are:

- the use of a well-established, validated and accurate objective measure of sitting (activPAL) as the reference measure, in contrast to most previous research which used measures of low movement (such as the ActiGraph) rather than postural sensors [8];
- the use of taxonomic systems [3, 8] to provide comparative validation between measures within one validation study, allowing extrapolation of the results to all self-report measures of sedentary time;
- the sample size (*n* = 700), which is larger than many validation studies published to date [8], and high compliance within the study (92% of participants agreeing to take part provided data for a full 7-day period).

The main limitation to this study is the lack of objective detection of waking time. The analysis relies on waking day data and this is ascertained using a sleep diary. While these are generally considered reliable and valid [23], they are not free of bias and error. Consequently, they may have degraded the quality and accuracy of the reference measure. Although automated methods to detect sleep show promise [24, 25], they do not currently offer a sufficient advantage over a sleep diary.

Finally, the results should be interpreted with care because they are based on a sample of older adults from three ongoing cohorts, only some of whom were employed, and might not be directly generalisable to self-report assessment in children or adult populations, for different cultural contexts, or in those perhaps less interested in their health than those who volunteer for repeat data collections within an ongoing research cohort. While the systematic approach taken in this comparative validation process should provide generalisable information for all self-report tool, replication of this process would provide definite proof of consistency of the findings.

### Future

The results show that it is unlikely that great improvements in accuracy can be gained by developing new questionnaires or adapting existing ones. The heteroscedasticity (the variability of a variable is unequal across the range of values of a second variable that predicts it) present in several of the self-report measures suggest that part of the error is not entirely random and might have some deterministic sources. This suggests that individual answers could be corrected with some calibration equations using respondent characteristics. However, a recent study found that calibrating a single item direct measure of total sitting measured on 183 blue-collar workers based on a prediction model using standard demographic information only improved accuracy by 10 to 30% [26]. Future research should certainly consider exploring calibration of data, however this may lead to overfitting the data or increased burden. Another potential route for improvement might be using adaptive testing and presenting type of assessment and recall period tailored to the individual respondent.

There is an increasing interest in studying SBs in more detail and context is seen as key [11], so more questionnaires using composite assessment are appearing [27]. These composite measures are a trade-off. They provide information about time spent sedentary in specific behaviours or domains and still enable an estimate of total sedentary time to be made. However, the subscales within these composite measures are never validated, so the quality of the information on time spent in specific domains or behaviours is really unknown. Additionally, as seen within this study, the measurement characteristics of these sums to assess total sedentary time are inferior to other type of assessments. In the future, specific validation of sub-scales to ascertain their individual validity should be performed against an appropriate reference measure other than total sedentary time.

## Conclusion

This systematic validation of 18 self-report measures of sedentary time (based on the TASST taxonomy) in 700 older adults, generally showed a large bias, large random error and low correlation with an objective measure of sedentary time. The type of assessment used in these measures had a much larger influence on measurement characteristics than the recall period. Overall, assessing the proportion of the day spent sitting using a visual analogue scale or a proxy measure such as TV time, demonstrated better measurement characteristics than any other type of measure. The results, in combination with the TASST taxonomy of self-report tools, enabled recommendations to be made for the choice of self-report measure to use in surveillance and epidemiological studies and provided correction factors for total sedentary time to enable comparison between studies and improve population estimates of average sedentary time.

## Additional files

Additional file 1: List of sedentary behavior questionnaires mapped on the TASST taxonomy. (PDF 285 kb)

Additional file 2: Bland and Altman plots. (PDF 439 kb)

Additional file 3: Self-report tools. (PDF 319 kb)
