# Supplementary material for: Systematic comparative validation of self-report measures of sedentary time against an objective measure of postural sitting (activPAL)
Source: Int J Behav Nutr Phys Act. 2018 Feb 26;15:21. doi: 10.1186/s12966-018-0652-x (PMC5828279; doi:10.1186/s12966-018-0652-x)
Supplement: Supplementary file 1 — List of sedentary behavior questionnaires mapped on the TASST taxonomy. (PDF 285 kb) [file 12966_2018_652_MOESM1_ESM.pdf]

Systematic comparative validation of self-reported measures of sedentary time using the TASTT taxonomy.

Additional file 1. List of sedentary behaviour questionnaires mapped on the TASST taxonomy

Table S3 Distribution of existing self-report tools for measuring SB in adults, classified on TASST domains of type of assessment and recall period, demonstrating those tested and recommended from the Seniors USP study.

Type of Assessment		Recall Period<br>Previous Day<br>*2.1*	 Previous Week<br>*2.2*	 Unanchored<br>*2.4*
Single Item	Direct: total sitting<br>*1.1.1*		*AusDiab*[2]<br>*IPAQ-L l7d*[2]<br>*IPAQ-S l7d*[2]<br>PASE	45Up-B<br>CFS<br>GPAQ<br>HUNT3<br>*IPAQ-L uw*[2]<br>*IPAQ-S uw*[2]<br>*PCSPa*[2]
Single Item	Direct: proportion<br>*1.1.1*			
Single Item	Proxy<br>*1.1.2*		*AusDiab*[2]	45Up-B<br>*ELSA*[2]<br>MLTPAQ<br>*NSWPAS*[2]<br>*SHS*[2]
Composite	Pattern<br>*1.2.1*			
Composite	Sum: of Behaviours<br>*1.2.2.1*	PAST<br>PAST-U	*45Up-F*[2]<br>*ALTS*[1]<br>*mod IPAQ-L*[2]<br>*MOST*[1]<br>*SIT-Q-7d*[2]	*LASA*[2]<br>mod NHANES<br>*SBQ*[2]
Composite	Sum: of Domains<br>*1.2.2.2*			

Data adapted from table 2, Dall PM, Coulter EH, Fitzsimons CF, Skelton DA, Chastin SFM, on behalf of the Seniors USP Team. The TAxonomy of Self-reported Sedentary behaviour Tools (TASST) framework for development, comparison and evaluation of self-report tools: content analysis and systematic review. BMJ Open 2017;7:e013844 [8].

Self-report tools were included if they assessed SB and were suitable for used in adult, up to a search in November 2016. Tools that had a longer recall period (TASST taxon *2.3*) have been excluded from this table, as these were not assessed in the Seniors USP study. The Seniors USP study used a questionnaire which assessed all types of assessment and recall periods, even those where previously published self-report tools were not available (see supplemental material 2)

[1] Tools where the Temporal Unit is not “Day” (TASST taxon *3.1*); [2] Tools where the Assessment Period is not “Not defined” (TASST taxon *4.5*). Tools in plain text (not italics) represent those with full TASST taxonomy was tested in the study. Shading indicates best type of assessment/recall period combinations based on small error and data loss (see figure 4). Diagonal hatching indicates those preferentially recommended to measuring total SB for surveillance studies (see figure 5).

45Up-B: 45 and Up study, baseline questionnaire; 45Up-F: 45 and Up study, follow up questionnaire; ACS2: American Cancer Society, Cancer Prevention Study cohort II; ALTS: Australian Leisure Time Sitting questionnaire; AusDiab: The Australian Diabetes Obesity and Lifestyle study; CCHS: Canadian Community Health Survey; CFS: Canadian Fitness Survey; CHAMPS: Community Health Activities Model Program for Seniors physical activity questionnaire; ELSA: English Longitudinal Study of Ageing; EPAQ2: European Prospective Investigation of Cancer (EPIC)-Norfolk Physical Activity Questionnaire; mod EQPAQ2: modified version of the EPIC-Norfolk Physical Activity Questionnaire; GPAQ: Global Physical Activity Questionnaire; HSE: Health Survey for England; HUNT3: Nord-Trøndelag Health Study 3; IPAQ-L l7d: International Physical Activity Questionnaire, Long version, last 7 days; IPAQ-L uw: International Physical Activity Questionnaire, Long version, usual week; mod IPAQ-L: modified version of the International Physical Activity Questionnaire, Long version; IPAQ-S l7d: International Physical Activity Questionnaire, Short version, last 7 days; IPAQ-S uw: International Physical Activity Questionnaire, Short version, usual week; LASA: Longitudinal Aging Study Amsterdam; MLTPAQ: Minnesota Leisure Time Physical Activity Questionnaire; MOST: Measuring Older adults' Sedentary Time questionnaire; NHANES: National Health and Nutrition Examination Survey; mod NHANES: modified version of the National Health and Nutrition Examination Survey; NHS2: Nurses Health Survey II; NIH-AARP DHS: National Institutes of Health – American Association of Retired Persons (NIH-AARP) Diet and Health Survey; NSWPAS: New South Wales Physical Activity Survey; PASE: Physical Activity Scale for the Elderly; PAST: Past-day Adults Sedentary Time questionnaire; PAST-U: Past-day Adults Sedentary Time questionnaire – University version; PCSpa: prospective cohort study (Spain); SBQ: Sedentary Behaviour Questionnaire; SHS: Scottish Health Survey; SIT-Q: SIT-Q; SIT-Q-7d: past seven day version of the SIT-Q; STAR-Q: Sedentary Time and Reporting Questionnaire; STAQ: Sedentary, Transportation and Activity Questionnaire.
