# Supplementary material for: Systematic comparative validation of self-report measures of sedentary time against an objective measure of postural sitting (activPAL)
Source: Int J Behav Nutr Phys Act. 2018 Feb 26;15:21. doi: 10.1186/s12966-018-0652-x (PMC5828279; doi:10.1186/s12966-018-0652-x)
Supplement: Supplementary file 2 — Bland and Altman plots. (PDF 439 kb) [file 12966_2018_652_MOESM2_ESM.pdf]

Systematic comparative validation of self-reported measures of sedentary time using the TASTT taxonomy.

Additional file 2.

Bland and Altman plots

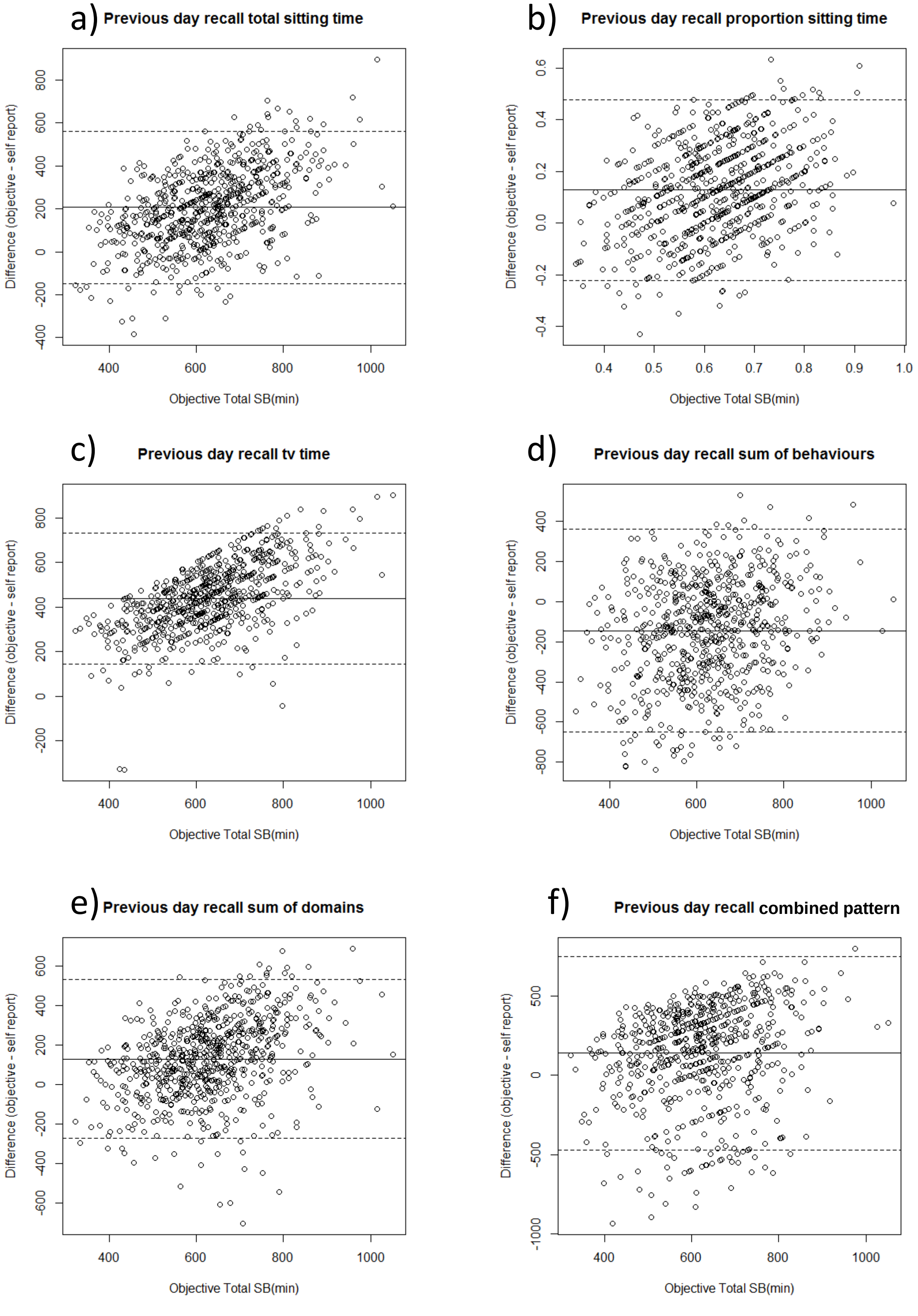

Figure S1: Bland and Altman plots for previous day recall self-reported tools of sedentary time

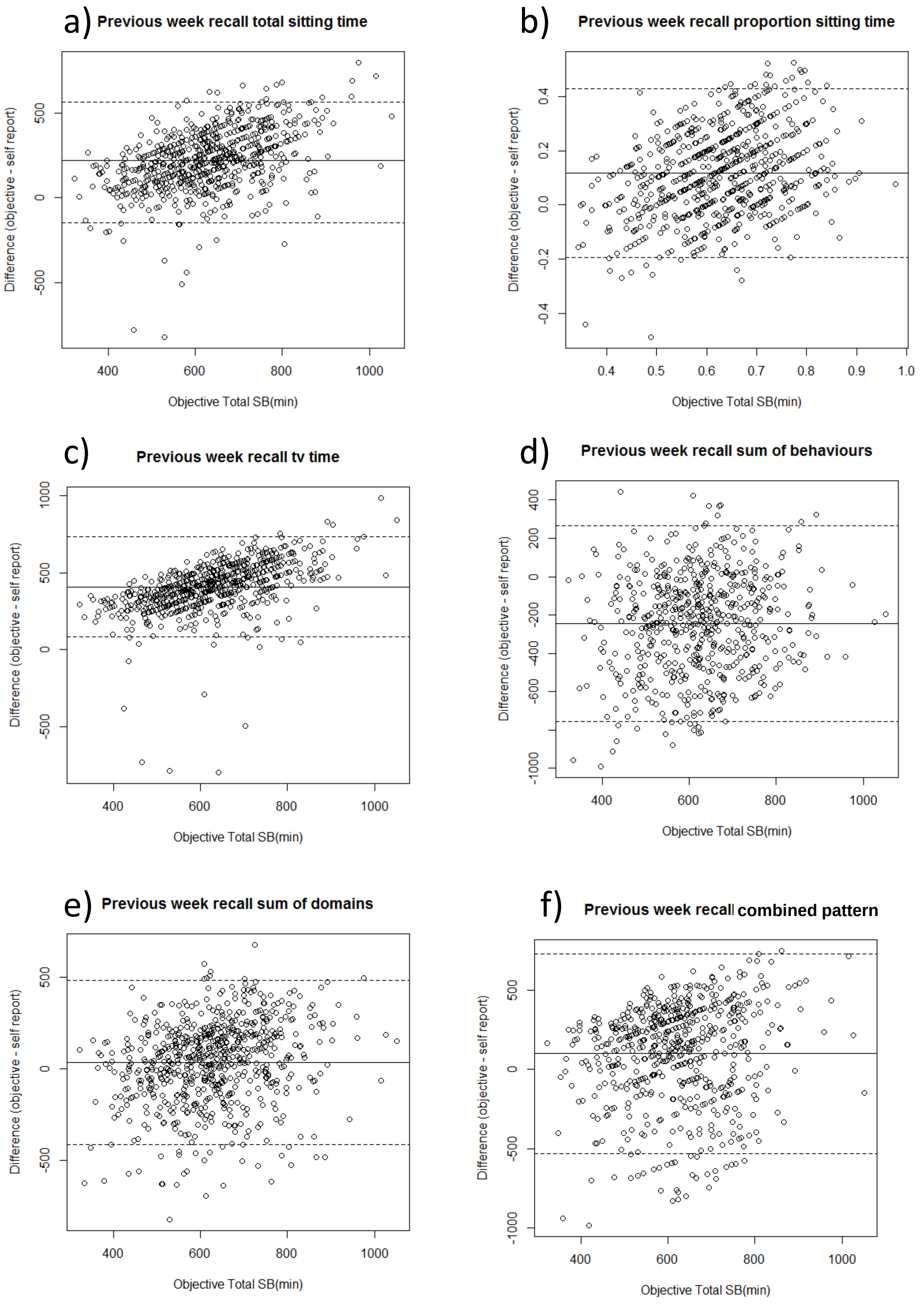

Figure S2: Bland and Altman plots for previous week recall self-reported tools of sedentary time

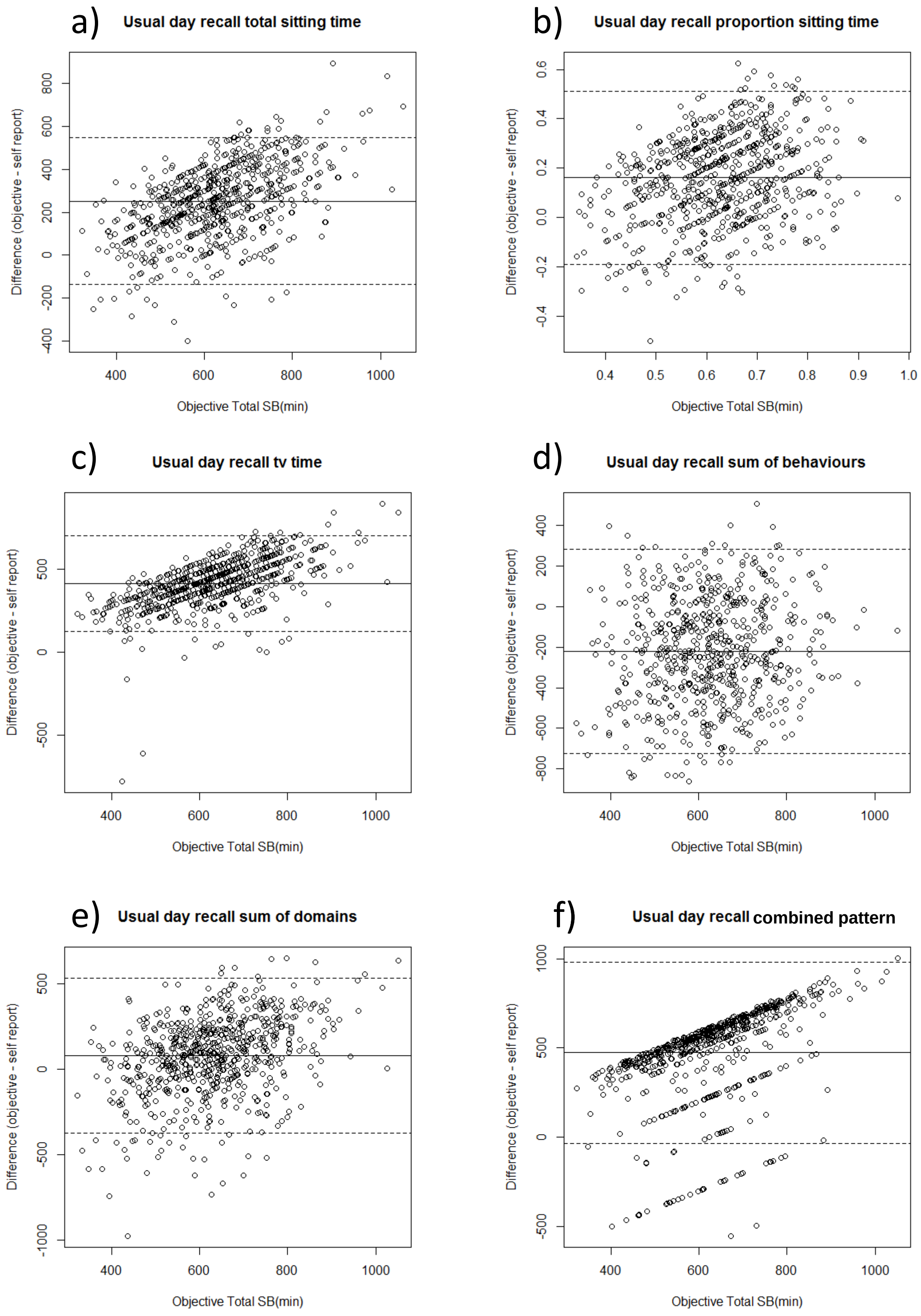

Figure S3: Bland and Altman plots for unanchored recall period (Usual day) self-reported tools of sedentary time
