# Supplementary material for: Systematic comparative validation of self-report measures of sedentary time against an objective measure of postural sitting (activPAL)
Source: Int J Behav Nutr Phys Act. 2018 Feb 26;15:21. doi: 10.1186/s12966-018-0652-x (PMC5828279; doi:10.1186/s12966-018-0652-x)
Supplement: Supplementary file 3 — Self-report tools. (PDF 319 kb) [file 12966_2018_652_MOESM3_ESM.pdf]

Systematic comparative validation of self-reported measures of sedentary time using the TASTT taxonomy.

Additional file 3.

Self-report tools

Self-reported tool

This document contains the six different self-reported tools for Taxon 1 of the TASTT framework used in the validation study.

These are presented here for previous day recall period (taxon 2.1 of the TASTT framework) only . Questions were identical for the other recall periods; previous week recall (taxon 2.2) and unanchored (usual day) (taxon 2.3). Only words referring to the recall period changed. “Yesterday” was replaced by “average day, in the last seven days ” for taxon 2.2 and “on a usual day” for taxon 2.3 .

Taxon 1.1.1 Direct measure

Total sedentary time

Please estimate how long in total you spent sitting yesterday?

(Please **write in**) ______ hours ______ minutes

Proportion of sedentary time

Yesterday, what proportion of the day did you spend sitting?

Please mark an X on the line.

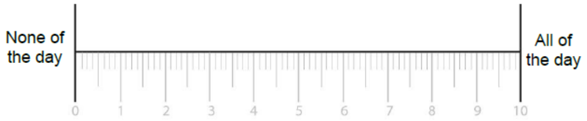

Taxon 1.1.2 Proxy measure

Please **write in** how much time you spent sitting and doing the following activities **yesterday**.

	Hours	Minutes
Watching television, video or DVDs.		

Taxon 1.2.1 Composite pattern

Yesterday, how many times did you sit down?

(Please **write in**) ______ times

X

Yesterday, when you sat down, how long was it before you usually got up again?

(Please **write in**) ______ hours ______ minutes

Taxon 1.2.1 Composite sum domains

Yesterday, how long did you sit at work (either paid or voluntary), or doing clerical tasks such as paying bills or filling in forms?

Please include tasks both inside and outside your home.

(Please **write in**) ______ hours ______ minutes

Yesterday, how long did you sit when you were at home?

(Please **write in**) ______ hours ______ minutes

Yesterday, how long did you sit while using transport (e.g. car, bus, train, tram or underground)?

Please include time spent sitting waiting for transport such as sitting at a bus stop.

(Please **write in**) ______ hours ______ minutes

Yesterday, how long did you sit during leisure activities outside your house?

Please include activities such as the cinema, eating at a restaurant, using the library, going to the theatre, religious practices, attending sport events, meeting friends at a coffee shop etc.

(Please **write in**) ______ hours ______ minutes

Taxon 1.2.1 Composite sum behaviours

Please **write in** how much time you spent sitting and doing the following activities **yesterday**.

	Hours	Minutes
Watching television, video or DVDs.		
Using a computer for leisure. (e.g. games, friends and family, internet, shopping)		
Read for leisure. (e.g. books, newspaper, magazine, kindle)		
Listening to music or radio.		
Doing a hobby. (e.g. knitting, crafts, jigsaws, puzzles, playing piano)		
Talking with friends or family. (e.g. talking in person or on the phone)		
Eating meals or snacks.		
Performing self-care tasks. (e.g. bathing, using toilet, dressing, brushing hair)		
Household tasks. (e.g. cleaning shoes, writing a list, ironing, folding)		
Taking a nap during the day or resting while doing nothing else.		

Yesterday, how long did you sit while using transport (e.g. car, bus, train, tram or underground)?

Please include time spent sitting waiting for transport such as sitting at a bus stop.

(Please **write in**) ____ hours ____ minutes

Yesterday, how long did you sit during leisure activities outside your house?

Please include activities such as the cinema, eating at a restaurant, using the library, going to the theatre, religious practices, attending sport events, meeting friends at a coffee shop etc.

(Please **write in**) ____ hours ____ minutes

Yesterday, how long did you sit at work (either paid or voluntary), or doing clerical tasks such as paying bills or filling in forms?

Please include tasks both inside and outside your home.

(Please **write in**) ____ hours ____ minutes
